# 

**Published:** 1892-03

**Authors:** 


ATLANTA

MEDICAL AND SURGICAL JOURNAL.

OLD SERIES VOL. XXVI. NEW SERIES VOL. IX.

### INDEX.

ORIGINAL COMMUNICATIONS.

Abortion, A New Method for the Management of (Harris), 135.
Abdominal Hysterectomy for Fibroids (Bizzell), 457.
Abortion, Treatment of, and some of the Complications Incident thereto (Crow), 276.
Abortion, What Shall We Do with the Uterus after (Moore), 321.
Accidental Wound of Femoral Artery (Dickson), 667.
Adenoid Vegetations of the Pharynx, a Frequent Cause of Deafness in Children, their Removal (Phillips), 329.
Amputation of the Thigh (Manley), 193.
Artificial Feeding of Infants (Shaw), 389.
Asthma, Treatment of (Mays), 663.
Atrophic Rhinitis (Roy), 262.
Cancer, The Cure of, by Mattei-ism, from the French of M. Louis Boucher, 334.
Cancer of Cervix, Concerning Value of Partial Operations in the Treatment of, from French of D. E. Monod, 75.
Carcimona, Extirpation of the Rectum for (Gaston), 129.
Cerebro-Spinal Hyperæmia, Case of (Moreland), 397.
Cholera, Treatment of (Atkinson), 708.
Cholera, History of in New Orleans (Jones), 465.
Clinical Observation on Continued Fever, etc. (Richardson), 523.
Clinical Reports Atlanta Medical College, 140.
Compatibility of Conservative and Aggressive Surgery (Gaston), 577.
Cretinism, Case of (Hagan), 705.
Cystitis (Murphy), 11.
Electricity as a Therapeutic Means in Lateral Curvature (Hagan), 394.
Empyema (O'Keef), 530.
Ether and Chloroform (Williams), 723.
Fever, Typho-Malarial (Duncan), 198.
Gynecology, What is (Kime), 270.
Hernia, Umbilical (Manley), 193.
Hemorrhage, Post-partum (Williams), 211.
Intestinal Injury, A Case of, Recovery (Johnstone), 344.
Idiosyncrasy, Peculiar, Treatment of (Bass), 74.
Laparotomies Performed during Past Year (Thos. Opie, Discussion by W. E. B. Davis), 18.
Laparotomies, Twenty-eight Consecutive (Hardon), 1.
Medical Education, Present Demand for Better (Grandy), 513.
Modern Electrical Methods (Massey), 535.
Myoma, Uterine (Manley), 193.
Os Uteri, A Case of Occlusion of (Davis), 342.
Ovarian Pregnancy, Case of (Lanphear), 590.
Peritonitis, Puerperal, The Surgical Treatment of (Davis), 257.
Pleurisy (McKee), 651.
Points Obtained in New York (Day), 715.

(1892-3.)

### INDEX.

Post-Partal Hemorrhage, Treatment of (Grynfelt), 385.
Present Status of Surgery of the Vermiform Appendix (Wyeth), 400.
Pneumonia, The Pathology and Treatment of (Johnson), 65.
Post-partum Hemorrhage (Williams), 211.
Puerperal Septicæmia, Recent Experiences with (Haggard), 206.
Relations of Bacteria to Disease (Blackford), 449.
"Ringworm" (Hutchins), 20.
Salpingitis, Treatment of (Goelet), 586.
Septicæmia, Puerperal, Recent Experiences with (Haggard), 206.
Shurley-Gibbes Treatment of Tuberculosis (Taylor), 593.
Skin Diseases, The Relation Between and the General Health (Hutchins), 346.
Supra-Pubic Cystotomy (White), 469.
Talipes-Equino-Varus, Treatment of (Barrier), 641.
Uterine Fibromata (Baldy), 656.

### SOCIETY REPORTS.

Allegheny County Medical Society, 471, 408, 537, 282.
American Association of Obstetricians and Gynecologists, 540.
Atlanta Society of Medicine, 26, 87.
Clinical Society of Maryland, 80, 157, 218, 278, 346, 730.
Discussion of Dr. Brown's Paper (S. S. and G. Ass'n), 36.
Georgia State Medical Association, 142.
Southern Surgical and Gynecological Association, 594, 669.

### CORRESPONDENCE.

Idiosyncrasy, A Peculiar Case of, 348.
Items of Medical Interest in New York, 44.
New York Letters, 91, 163, 225, 349, 285.

### EDITORIAL.

Amick Cure, 748.
American Medical Association, 280.
Atlanta Polyclinic, 749.
Bearing of Recent Biological Researches on the Practice of Medicine and Surgery, 50.
Bichloride of Gold Industry, 101.
Change in The Journal, 97.
Cholera, 422.
Closing of the Medical Schools, 56.
Cœliotomy, not Laparotomy, 551.
Death of Dr L. P. Kennedy, 296.
Europhen, 55.
Forty-third Annual Session of the Medical Association of Georgia, 171.
Gall-Bladder Surgery, 230.
Higher Medical Education, 356.
Ideal Physician, 489.
Keely in the Pulpit, 236.
Medical Association, 102.
Medical Examiners Bill, 611, 744.
Medical Journals and Advertising, 550.
Mitchell-Ward Case, 420.
Money Value Again, 424
Money Value of Medical Attendance, 298.
Nitroglycerine, Dose of, 487.
Present Status of the Medical Colleges of Georgia, 353.
Rowell, Geo. P. & Co., etc., 486.
Salts of Strontium, 237.
Southern Medical College Association, 615.
Social Evil, 617.
Some Legislation that We Need, 546.
Squeal from the Eclectics, 679.
The Grady Memorial Hospital, 168.
Treatment of Tuberculosis by Guaiacol, 98.
Treatment of Anæmia by Arsinite of Copper, 100.
Typhoid Fever, Treatment of, etc., 488.
Vivisection, 746.
Why "Heart Failure," 234.

### CONTRIBUTORS TO VOL. IX.

Atkinson, I. E., M. D., Baltimore.
Baldy, J. M., M. D., Philadelphia.
Barrier, C. W., M. D., Columbus, Ga.
Bass, J. L., M. D., Brewton, Ala.
Bizzell, B. W., M. D., Atlanta.
Blackford, Chas. M., M. D., Lynchburg.
Crow, W. A., M. D., Atlanta.
Davis, W. E. B., M. D., Birmingham.
Davis, Wm. L., M. D., Albany, Ga.
Day, W. C., M. D., Winchester, Ills.
Dickson, E., M. D., Coal Creek, Tenn.
Duncan, J. W., M. D., Atlanta.
Gaston, J. McF., M. D., Atlanta.
Goelet, Augustin H., M.D., New York.
Grandy, Luther B., M. D., Atlanta.
Grynfeit, Prof., France.
Hagan, Hugh, M. D., Atlanta.
Hardon, Virgil O., M. D., Atlanta.
Harris, Chas. H., M. D., Cedartown, Ga.
Haggard, W. D., M. D., Nashville.
Hutchins, M. B., M. D., Atlanta.
Johnson, J. Clarence, M. D., Atlanta.
Johnston, F. C., M. D., Richland, Ga.
Jones, Joseph, M. D., LL. D., New Orleans.
Kime, R. R., M. D., Atlanta.
Lanphear, Emory, M. D., Ph. D., Kansas City, Mo.
Manley, Thos. H., M. D., New York.
Massey, G. Belton, M. D., Philadelphia.
Mays, Thos. J., M. D., Philadelphia.
Moore, K. P., M. D., Macon.
Moreland, A. C., M. D., Atlanta.
Murphey, C. E., M. D., Atlanta.
O'Keef, P., M. D., Oconto, Wis.
Phillips, S. Latimer, M. D., Savannah.
Richardson, E. H., M. D., Atlanta.
Roy, Chas. D., M. D., Atlanta.
Russell, Wm. L., M. D., New York.
Shaw, Chas. S., M. D., Pittsburg.
Taylor, H. Longstreet, M. D., A. M., Asheville, N. C.
White, Jas. S., M. D., Columbia, Ky.
Williams, Roger, M. D., Pittsburg.

